# Adenomyoma of the round ligament of the uterus in the inguinal canal: a case report

**DOI:** 10.1093/jscr/rjaf421

**Published:** 2025-09-24

**Authors:** Linhan Dai, Jin Wang, Yuhan Chen, Lvjia Cheng, Xiaoshen Zhang, Wenbin Zhang

**Affiliations:** The First Affiliated Hospital of Jinan University, 510630 Guangzhou, Guangdong Province, PR China; The First Affiliated Hospital of Jinan University, 510630 Guangzhou, Guangdong Province, PR China; The First Affiliated Hospital of Jinan University, 510630 Guangzhou, Guangdong Province, PR China; The First Affiliated Hospital of Jinan University, 510630 Guangzhou, Guangdong Province, PR China; The First Affiliated Hospital of Jinan University, 510630 Guangzhou, Guangdong Province, PR China; The First Affiliated Hospital of Jinan University, 510630 Guangzhou, Guangdong Province, PR China

**Keywords:** inguinal hernia, round ligament, uterine adenomyoma

## Abstract

Adenomyoma originating from the round ligament of the uterus represents a remarkably uncommon pathological entity. Considering the anatomical course of the round ligament through the inguinal canal, pathological lesions in this structure may clinically present as pelvic or inguinal masses, thereby requiring differentiation from a broad spectrum of alternative pathological conditions. Of particular clinical significance, adenomyoma-associated manifestations causing inguinal hernia remain exceedingly rare, with definitive diagnostic confirmation contingent upon histopathological verification. We herein present a case of primary round ligament adenomyoma localized within the inguinal canal, with the objective of enhancing clinical awareness and providing evidence-based guidance and optimizing diagnostic and therapeutic decision-making in comparable clinical scenarios.

## Introduction

Adenomyoma, a localized nodular form of adenomyosis, is characterized by the presence of ectopic endometrial-like glands and stroma within the myometrium. This gynecological condition predominantly affects women during their reproductive years. Ultrasonographic diagnostic sensitivity for this condition ranges from 20.9% to 34% [1]. Clinically, adenomyoma manifests as menorrhagia, prolonged menstrual duration, severe dysmenorrhea, or chronic pelvic pain. Although its exact pathogenesis remains unclear, several theories have been proposed—including invasive endometrial tissue growth, fetal developmental remnants, postpartum inflammatory changes, stem cell origins, and immune or inflammatory factors—all of which are related to estrogen levels. Consequently, the condition often regresses following menopause due to declining estrogen levels [1]. Extrauterine adenomyomas, while rare, exhibit a characteristic anatomic distribution predominantly localized to the pararectal space, ovaries, and broad ligaments, and retroperitoneal structures, with less frequent involvement of the round ligaments, uterine serosa, and pelvic sidewall [2]. Embryologically, the round ligament of the uterus is homologous to the gubernaculum testis. Tumors originating from the round ligament are uncommon, with uterine fibroids constituting the predominant pathological type [3–9]; adenomyomas of this structure are exceptionally rare [10].

## Case presentation

A 51-year-old female presented on 23 October 2024, with a chief complaint of a persistent left inguinal mass for over 3 years. The mass, initially ~2–3 cm in diameter (jujube-sized), was noted to protrude during standing or exertion and exhibited spontaneous reduction in the supine position, without associated pain or discomfort. No medical intervention was pursued initially. Two years post-onset, the mass became irreducible but demonstrated partial reduction following unspecified traditional Chinese medicine therapy at a local hospital. Progressive enlargement to ~5 cm (egg-sized) subsequently occurred, with persistent irreducibility even in the supine position. The patient underwent menopause at age 50, with no reported postmenopausal abdominal pain, vaginal bleeding, or abnormal discharge. Menstrual history revealed regular 25- to 28-day cycles with 7-day flow duration; no fertility impairments or family history of similar conditions were documented.

**Figure 1:**
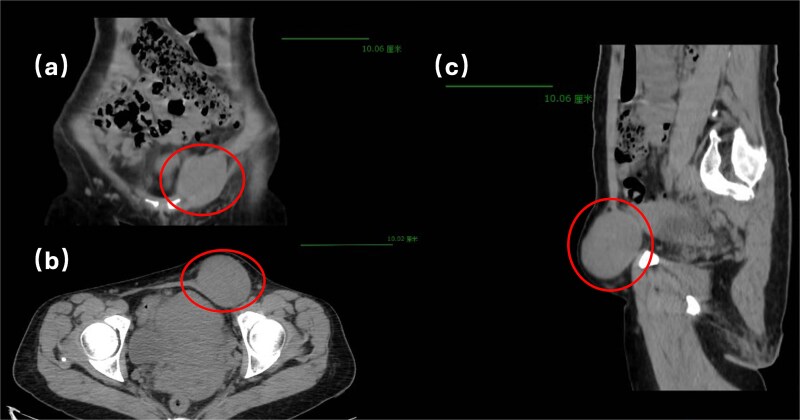
Pelvic computed tomography.

**Figure 2:**
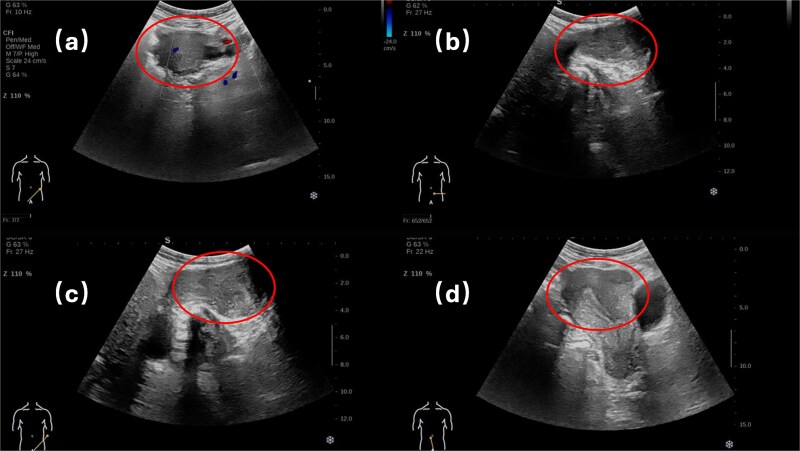
Superficial organ ultrasound and transvaginal ultrasound.

Physical examination revealed a soft, non-tender abdomen without hepatosplenomegaly or shifting dullness; normal bowel sounds were auscultated. A 5 × 5 cm firm, well-circumscribed mass with smooth surface contour and mild tenderness was palpated in the left inguinal region, demonstrating irreducibility and resistance to manual compression. Preoperative imaging modalities—contrast-enhanced computed tomography (Fig. 1) and grayscale ultrasonography (Fig. 2)—suggested a provisional diagnosis of uterine fibroid.

**Figure 3:**
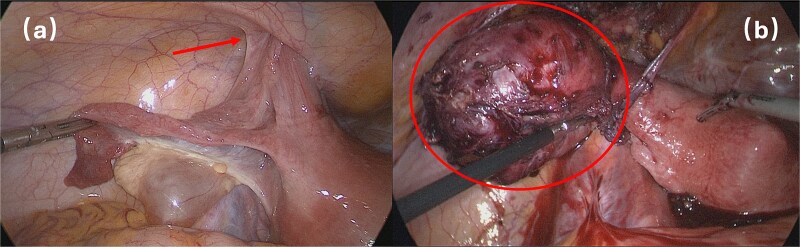
Surgical intervention.

**Figure 4:**
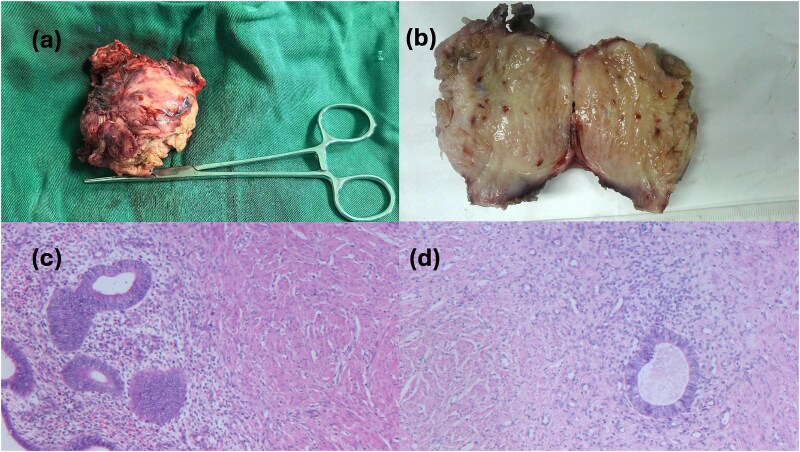
Postoperative gross pathology and HE staining.

Surgical intervention comprised en bloc excision of the mass with the affected round ligament segment, followed by laparoscopic tension-free mesh hernioplasty (Fig. 3). Histopathological analysis confirmed uterine adenomyoma (Fig. 4). The patient was discharged on postoperative Day 5 following an uneventful recovery.

## Discussion

In this case, the patient exhibited no documented gastrointestinal symptoms—specifically, the absence of abdominal pain, bloating, nausea, vomiting, or alterations in bowel habits—throughout the disease course. Premenopausal menstrual cycles were characterized by regular 25- to 28-day intervals with physiological blood loss. Although adenomyotic lesions are typically estrogen-dependent and tend to regress after menopause, some patients may still experience subtle postmenopausal manifestations such as vague pelvic pressure or discomfort due to residual tissue changes [1]. Notably, this patient demonstrated complete resolution of gynecological symptoms postmenopause, with neither pelvic pain nor abnormal vaginal bleeding or discharge observed. Contrary to expected regression patterns, the left inguinal mass exhibited progressive enlargement, culminating in irreducibility necessitating surgical intervention. Although preoperative imaging likely suggested an incarcerated hernia of uterine fibroid origin, definitive histopathological diagnosis confirmed uterine adenomyoma.

We evaluated the following two potential mechanisms for the development of the incarcerated hernia in this patient:

1. Primary origin: An adenomyoma developing within the inguinal canalicular segment of the round ligament in the inguinal canal, with gradual enlargement over time.
2. Secondary herniation: An adenomyoma originating from the pelvic segment of the round ligament that herniated into the inguinal canal due to increased intra-abdominal pressure, subsequently becoming entrapped as the mass expanded.

Through multidisciplinary consultations with gynecologic and gastrointestinal surgical specialists, we proposed that a primary origin within the inguinal canal is more plausible than the secondary herniation process.

Adenomyoma is defined by the pathological presence of endometrial glands and stroma within the myometrium. Extrauterine manifestations of adenomyoma are exceptionally rare, with predominant localization observed in the rectovaginal septum, ovaries, and broad ligaments. Secondary sites include the uterine round ligaments, pelvic peritoneum, and lateral pelvic wall. The anatomical course of the uterine round ligament through the inguinal canal contributes to diagnostic challenges in adenomyomas arising from this structure. Given the rarity of round ligament adenomyomas, clinical management necessitates a comprehensive differential diagnosis encompassing inguinal or femoral hernias, uterine leiomyomas, leiomyosarcomas, endometriotic implants, and congenital round ligament pathologies. Preoperative characterization remains challenging due to nonspecific imaging features, with definitive diagnosis requiring histopathological confirmation. Complete surgical excision represents the therapeutic cornerstone, achieving both diagnostic and curative objectives. By delineating a rare clinical entity, this case report enhances clinical awareness, provides evidence-based guidance, and optimizes decision-making for similar cases, thereby contributing to the existing literature.
